# Practical considerations for engaging staff in resource-constrained healthcare settings in implementation research: A qualitative focus group and consensus building study

**DOI:** 10.1017/cts.2025.29

**Published:** 2025-03-26

**Authors:** Kelly A. Aschbrenner, Callie Walsh-Bailey, Meagan C. Brown, Tanveer Khan, Travis P. Baggett, Salene M.W. Jones, Douglas E. Levy, Lydia E. Pace, Jonathan P. Winickoff

**Affiliations:** 1 Department of Psychiatry, Dartmouth Hitchcock Medical Center, Lebanon, NH, USA; 2 Department of Medical Social Sciences, Division of Implementation Science, Northwestern University Feinberg, School of Medicine, Chicago, IL, USA; 3 Kaiser Permanente Washington Health Research Institute, Seattle, WA, USA; 4 University of Washington, Department of Epidemiology, School of Public Health, Hans Rosling Center for Population Health, Seattle, WA, USA; 5 Harvard Street Neighborhood Health Center, Dorchester, MA, USA; 6 Division of General Internal Medicine, Massachusetts General Hospital, Boston, MA, USA; 7 Boston Health Care for the Homeless Program, Program Institute for Research, Quality & Policy Boston, MA, USA; 8 The Mongan Institute Health Policy Research Center, Massachusetts General Hospital, Boston, MA, USA; 9 Harvard Medical School, Boston, MA, USA; 10 Fred Hutchinson Cancer Center, Seattle, WA, USA; 11 Brigham and Women’s Hospital, Boston, MA, USA; 12 Division of General Academic Pediatrics, Mass General for Children, Boston, MA, USA

**Keywords:** Implementation research, resource-constrained healthcare settings, inner settings, staff, organizations, epis implementation framework, pragmatic research methods

## Abstract

**Background:**

The primary purpose of this study was to assess perceived burdens and benefits of participating in implementation research among staff employed in resource-constrained healthcare settings. Another objective was to use findings to generate considerations for engaging staff in research across different phases of implementation research.

**Methods:**

This qualitative focus group and consensus building study involved researchers affiliated with the National Cancer Institute Implementation Science Centers in Cancer Control program and nine Community Health Centers (CHCs) in Massachusetts. Six focus groups (n = 3 with CHC staff; *n* = 3 with researchers) assessed barriers and facilitators to staff participation in implementation research. During consensus discussions, we used findings to develop considerations for engaging staff as participants and partners throughout phases of implementation research.

**Results:**

Sixteen researchers and 14 staff participated in separate focus groups; nine researchers and seven staff participated in separate consensus discussions. Themes emerged across participant groups in three domains: (1) influences on research participation; (2) research burdens and benefits; and (3) ways to facilitate staff participation in research. Practical considerations included: (a) aligning research with organizational and staff values and priorities; (b) applying user-centered design to research methods; (c) building organizational and individual research capacity; and (d) offering equitable incentives for staff participation.

**Conclusions:**

Engaging staff as participants and partners across different phases of implementation research requires knowledge about what contributes to research burden and benefits and addressing context-specific burdens and benefits.

## Introduction

Clinical and administrative staff employed in the inner context of healthcare organizations are key informants about contextual factors influencing implementation as noted in prominent implementation science frameworks [1,2]. Furthermore, these staff are often key decision makers and/or deliverers of the evidence-based interventions (EBIs) that implementation research seeks to support. Yet, there are significant challenges to recruiting staff in healthcare settings as participants and partners in research, including gaining entry to recruit in a given setting, reaching potential staff participants, assessing staff’s willingness or ability to participate in research, and coordinating research activities with those who agree to participate [3,4]. These barriers are particularly salient for staff working in resource-constrained healthcare settings that experience financial pressures, underdeveloped infrastructures, and human resource limitations [5]. Staff have reported barriers to research participation such as lack of dedicated time to participate in research and concerns about loss of productivity or income while engaging in research [6]. These barriers to participation in research may result in unintended inequities with respect to the perspectives and experiences represented in implementation research and ultimately decisions made based on research findings.

Engaging healthcare staff who possess timely and contextually rich knowledge about their local practice settings in research is crucial for integrating and sustaining EBIs in practice. Calls for community engagement approaches in implementation science have emphasized the need to engage local knowledge and expertise, promote authentic relationships, and build community and researcher capacity (e.g., bidirectional knowledge exchange, skills, and experience) [7,8]. Using a community-engaged approach, clinical and administrative healthcare staff have been participants and partners in implementation studies [9–13]. However, less attention has been given to the practical aspects of how to address barriers and leverage facilitators to engage staff in research activities, which is particularly relevant in resource-constrained healthcare settings.

The current study was motivated in part by our team’s experiences conducting implementation research in partnership with Community Health Centers (CHCs) [13–15] and the limited research literature offering practical guidance for conducting research with staff working in resource-constrained healthcare settings. CHCs are the primary care safety net for uninsured and low-income individuals in the in the USA [16]. Staff working in CHCs have an insider’s view of gaps in healthcare delivery and health inequities that can be critical for informing implementation research. From a strengths-based perspective [17], CHC staff are poised to innovate and develop creative, frugal solutions that capitalize on available resources, which can offer valuable learnings across implementation settings. The primary purpose of the present study was to assess perceived burdens and benefits of participating in implementation research among staff employed in resource-constrained healthcare settings. Another objective was to use findings from the focus groups to identify considerations for engaging staff in research across different phases of implementation research.

## Methods

### Setting and design

This study was led by a team from the Implementation Science Center in Cancer Control Equity (ISCCCE), which is funded by the National Cancer Institute as one of seven centers within the Implementation Science Centers in Cancer Control (ISC^3^) program nationwide [18]. The ISC^3^ seeks to enhance the capacity of researchers, practitioners and communities to apply implementation science approaches, methods, and measures. ISCCCE is a partnership among the Harvard T.H. Chan School of Public Health, the Kraft Center for Community Health at Massachusetts General Hospital, and the Massachusetts League of Community Health Centers (MLCHC), a Primary Care Association that provides support and technical assistance to CHCs across Massachusetts. ISCCCE, in collaboration with the MLCHC, has partnerships with a network of 30 CHCs where community engaged research in implementation science is conducted [19].

Our research team collaborated with the MLCHC to purposively recruit CHC staff encompassing diverse roles within the organization, including Chief Operating Officer, Director of Quality Improvement, Director of Operations, Population Health Manager, Quality Improvement Manager, and Community Health Worker/Medical Interpreter. In addition, we recruited researchers from the ISC^3^ network as study participants. An advisory committee of ISC^3^ researchers helped to shape and refine the methods used and the practical considerations identified in this study. The Harvard Longwood Campus IRB, Mass General Brigham IRB, and Dartmouth Health Human Research Protection Program IRB independently approved the study protocol. The Consolidated Criteria for Reporting Qualitative Research was used to ensure compliance with reporting standards for qualitative research.

### Recruitment procedures

Our research team used purposive and convenience sampling [20] to recruit CHC staff and ISC^3^ researchers to participate in separate one-time 60-minute online focus groups. Eligible individuals were 18 years of age or older, spoke English, and were a) employed in a leadership or staff role at a partnering CHC within the ISCCCE network or b) a researcher affiliated with the ISC^3^ program with experience conducting research in resource-constrained healthcare settings. To recruit CHC participants, MLCHC leadership sent an email to employees at partnering CHCs advertising the study. In addition, the research team advertised the study to CHC staff participating in quarterly implementation learning community sessions conducted by ISCCCE. Our research team sent an email to 17 CHC staff who expressed interest in the study with information that included study procedures, anticipated time commitment, and incentives. Sixteen of the 17 CHC staff who expressed an interest in the study agreed to participate. Among those who participated in the focus groups, we recruited participants for 1-2 consensus discussions to identify practical considerations for engaging staff in research based on themes from the focus groups. CHC staff participants were compensated with a $100 gift card for participating in a one-time online focus group and $50 for each of two consensus discussions. To recruit ISC^3^ researchers, an ISC^3^ administrator sent an email with information about the study to listservs used to communicate relevant program information. The exact number of individuals on the listserv was not known by our research team. Our research team sent a follow-up email to 14 researchers who expressed interest in the study in response to the listserv announcement with information that included study procedures, anticipated time commitment, and incentives. All 14 researchers who received an email from our study team agreed to participate in the study. Similar to CHC participants, researchers were compensated with a $100 gift card for participating in a one-time focus group interview and $50 for each consensus discussion.

### Focus groups

Six focus groups (n = 3 with CHC staff; *n* = 3 with researchers) lasting 60 minutes each were conducted online between February and April 2024. The lead author drafted the interview guides that were reviewed by members of the advisory committee who provided feedback used to refine the interview questions. The final focus group topic guides assessed what influenced staff participation in research, burdensome aspects of research participation, benefits of research participation, and ways to reduce the burden of participating in research. Similar topic guides were used to ask questions and facilitate discussion in the CHC staff and ISC^3^ researcher focus groups. The lead investigator, experienced in qualitative research methods, conducted the focus group via Zoom with technical support from the study coordinator. All six focus groups were audio recorded and transcribed. Immediately following each focus group, the study coordinator sent an email to participants with a link to an online demographic survey that assessed individual level characteristics. Descriptive statistics were used to summarize and describe the demographic data.

We used an established team-based approach to coding the qualitative data [21]. At the beginning of the coding process, the lead investigator read through each of the six focus group transcripts and created a separate preliminary codebook for each participant type (i.e., CHC staff and ISC^3^ researchers) with codes aligned with the semi-structured topic guides. The codebooks included definitions for each *a priori* code and space for new codes that emerged during data analysis. The lead investigator and a second research team member independently applied the preliminary codebooks to code the transcripts using NVivo version 14 (Lumivero, Inc.) [22]. The two researchers met to discuss the application of the codebooks and then refined the codes and re-coded the transcripts applying an updated codebook in an iterative process until they arrived at a final set of codes for each participant type. The lead investigator identified preliminary themes across the codes, discussed them with the second researcher and the advisory committee who helped to interpret and finalize the themes [21].

### Consensus meetings

Consensus meetings were held with CHC staff participants and ISC^3^ researchers separately to reach agreement on a set of practical considerations for engaging staff employed in resource-constrained healthcare settings in implementation research. We convened separate consensus meetings with CHC staff and ISC^3^ researchers because we wanted to ensure the staff felt comfortable sharing feedback on the practical considerations informed by the focus group data. The consensus meetings were convened by the lead researcher via Zoom in four separate 60-minute meetings that included seven CHC staff and nine ISC^3^ researchers, respectively. The facilitator used principles from deliberative dialogue to moderate the consensus discussions. The deliberative dialogue process guides participants to seek a shared understanding of problems and to search for possible solutions focusing on logic and reason in an open and honest conversation that is grounded in both data and personal experiences [23].

**Figure 1.**
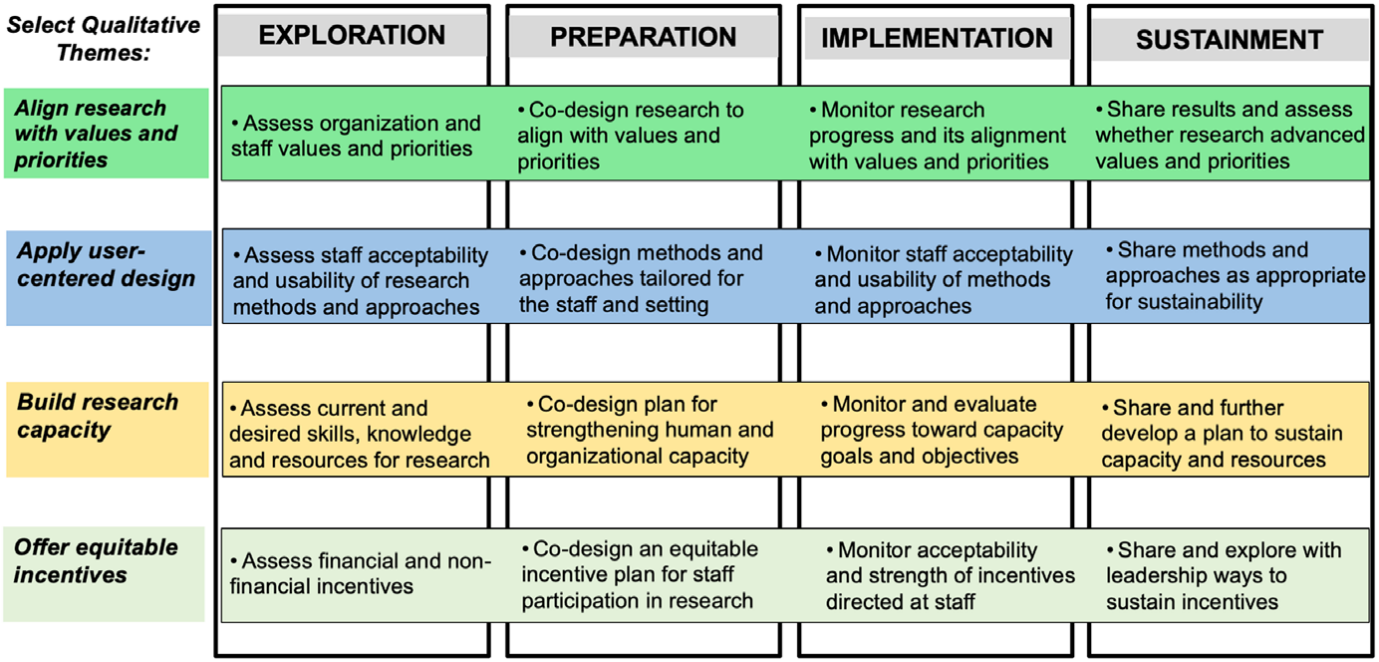
Practical considerations for promoting engagement in research among staff in the inner context mapped onto the exploration, preparation, implementation, and sustainment (EPIS) framework.

Prior to the consensus meetings, the lead researcher circulated a draft of practical considerations for engaging staff in research informed by the qualitative themes and mapped onto the Exploration, Preparation, Implementation and Sustainment (EPIS) framework, a multilevel, four phase model of the implementation process [24,25] (Figure 1). Participants were asked to critically review the figure and prepare to discuss feedback during the consensus meetings. The study team made minor revisions to the figure based on the feedback. Both participant groups suggested the study team develop scenario-based examples to illustrate how principles from the themes mapped to the EPIS model could be enacted in real world practice. The primary purpose of the scenarios was to provide detailed, learner-centered hypothetical examples of addressing challenges and leveraging facilitators to engaging healthcare staff as participants and/or partners in research. Our team circulated drafts of scenario-based examples to CHC staff and researchers for review and feedback which informed a final set of four scenarios (Supplemental Files 1-4).

## Results

### Focus groups

Sixteen of the 17 CHC staff who accepted our invitation participated in a focus group, representing nine different CHCs. Among the CHC staff participants, seven agreed to participate in at least one consensus discussion. All 14 researchers who accepted our invitation participated in a focus group. Among the researcher participants, nine agreed to participate in at least one consensus discussion. The background characteristics of the CHC staff participants and researcher participants are presented in Tables 1 and 2, respectively. The proportion of CHC staff participants who identified as racial and ethnic minorities was similar to the demographics of CHC patients reported nationally [26], while fewer ISC^3^ researcher participants identified as racial and ethnic minorities.

**Table 1.** Background characteristics of community health center (CHC) staff (N = 16)

Characteristics	*No. of participants*	*%*
*Age (years)*		
18-29	3	18.8
30-39	4	25
40-49	6	37.5
50-59	1	6.25
60 or older	2	12.5
*Sex*		
Male	1	6
Female	15	94
*Race* ^a^		
White	9	56.3
Black	4	25
American Indian or Alaska Native	2	12.5
Asian	1	6.25
Some other race	1	6.25
*Hispanic or Spanish Origin*		
Yes	4	25
*Educational level*		
High school degree or equivalent (e.g., GED)	2	12.5
Some college but no degree	1	6.25
Associate’s degree	2	12.5
Bachelor degree	3	18.8
Graduate degree	8	50
*Staff Position at Community Health Center* ^b^		
Director of Quality Improvement	2	12.5
Population Health Manager	2	12.5
Community Health Worker	5	31.3
Physician	1	6.3
Clinical Project Manager	1	6.3
Registered Nurse	1	6.3
Administrative Manager	1	6.3
Social Determinants of Health Program Manager	1	6.3
Clinical Director, COVID Response	1	6.3
Director of Substance Use Services	1	6.3
Patient Navigator	1	6.3
Program Manager	2	12.5
*Level of Experience with Research*		
High level of experience (participated in more than 3 projects)	5	31.3
Moderate level of experience (participated in 2-3 projects)	9	56.3
Average level of experience (participated in at least one project)	1	6.3
Low level of experience (have not participated in a project but familiar with projects taking place at the centre)	1	6.3
No experience with research at the centre	0	0

a One participant reported two races.

b Three participants reported two roles.

**Table 2.** Background characteristics of implementation science centers in cancer control (ISC^3^) researchers (N = 14)

Characteristics	*No. of participants*	*%*
*Age (years)*		
18-29	0	0
30-39	5	35.7
40-49	6	42.9
50-59	3	21.4
60 or older	0	0
*Sex*		
Male	3	21.4
Female	11	78.6
*Race* ^a^		
White	11	78.6
Black	3	21.4
American Indian or Alaska Native	0	0
Asian	1	7.14
Some other race	0	0
*Hispanic or Spanish Origin*		
Yes	1	7.14
*Educational level*		
Graduate degree	14	100
*Investigator Stage*		
Early stage investigator	5	35.7
New investigator	1	7.14
Early established investigator	1	7.14
Established investigator	3	21.4
Member of a research team in a role other than investigator	4	28.6

a One participant reported two races.

The qualitative codes for both participant types and themes across participant types are presented in Table 3. Since there were many similarities and few differences in responses from CHC staff and researcher participants to the focus group topic guide questions, we combined themes across participant types with illustrative quotes in the section below.

**Table 3.** Qualitative codes for community health center (CHC) staff and researcher participants and themes across participant types

CHC staff participants focus groups (n = 3)	Researcher participants focus groups (n = 3)	
*Q1. What factors influence CHC* ^a^ *staffs’ decision to participate in research?*		
**Codes**	**Codes**	**Themes**
Organizational priorities and staff personal and professional interests	Organizational mission, priorities, and values	1) Organizational and individual priorities and values 2) Impact on patient care delivery and outcomes 3) Incentives 4) Time and resource demands
Potential impact on patient care/outcomes	Potential impact on patient relationships	
Incentives for research participation	Incentives and compensation	
Time involved in research	Time and logistics	
Capacity building opportunities	Availability of perceived expertise	
Opportunity to partner with researchers	Return on investment	
Represent staff perspectives/experiences	Supervisor or leadership support	
	Who makes the request	
	Competing demands	
*Q2. What are burdensome aspects of research for CHC staff participation?*		
Adds to workload	Adds to workload	1) Adding to workload 2) Time burden 3) Limited capacity and resources
Cognitive burden	Burden of tasks	
Time and effort involved in research	Time and effort involved in research	
Limited capacity and resources for research	Data collection	
Engaging individuals vs. organizations	Emotional burden	
Language barriers	Stopping/changing behaviors, workflows	
Limited value added research activities		
*Q3. What are the benefits of research for CHC staff?*		
Impact on healthcare and patient outcomes	Capacity building	1) Improve healthcare delivery and outcomes 2) Staff recruitment and retention 3) Networking and relationship building
Learning what works	Improve workflow and performance	
Physician and provider recruitment	Additional resources	
Networking and relationship building	Recruit and retain staff	
	Networking and relationship building	
*Q4. What are ways to make it easier for CHC staff to participate in research?*		
Communicate purpose and value of staff participation in research	Be clear about research process and what to expect	1) Transparency about the research process 2) Organizational and individual capacity building 3) Apply user- centered design 4) Offer equitable incentives for staff 5) Bring more joy into research work
Engage staff as partners in early phases of research	Address organizational needs and solve problems	
Capacity building	Capacity building	
Offer appealing Incentives	Equitable compensation for research staff	
Make research fun and enjoyable	Integrate joy into research methodologies	
Use multilingual data collection methods	Employ a diverse research team	
Offer drop-in opportunities to join research	Tailor methods/approaches to the setting	
Include staff in a variety of roles in research	Draw on methods from other fields	
	Embed research in the health center	

a CHC = community health center.

#### Influences on staff participation in research

We began the focus groups by asking CHC staff and researcher participants to share their thoughts about what positively and negatively influences staff participation in research. Four themes emerged from the focus group data across participants types:

1. **Organizational and individual priorities and values**. Participants spoke of the importance of aligning research with organizational and individual priorities and values. CHC staff emphasized that both professional and personal interests in research topics influence their decision to participate in research. As one CHC staff participant commented:
*One of the things that can influence me to participate in research is when they’re looking at something that I am interested in, whether it’s for my work or personal life. For example, we’re doing research on breast cancer awareness, and I want to be involved in that because that’s how I can help my patients get their screenings done.*

1. **Impact on patient care delivery and outcomes.** Participants discussed how CHC staff were motivated to participate in research by the potential positive impact of research on patient care delivery and outcomes. As one CHC staff commented:
*One of the reasons why I participated in the last project is it allowed me to look at the whole implementation guide with a different perspective. And that meant learning something new because sometimes there’s complacency that develops within yourself and within your team.*

1. **Incentives.** Participants identified incentives as a factor that influenced staff participation in research. Specifically, researcher participants emphasized the importance of offering incentives to individual staff participants (vs. the entire organization). As one researcher noted:
*I would added compensation, which has been a mixed experience for us, where some health centers don’t allow individuals to be compensated, just at the center level. But I do think that acknowledging some kind of compensation, even if it is just a token of what the burden has been of that research, has been really important.*

1. **Time and resource demands.** Participants identified staff time and availability to participate in research as an influence on participation in research. In addition, participants emphasized employee skills as a resource often needed to conduct research. As one researcher noted:
*I will also say the complexity of whatever it is that we’re asking and the availability of the skills that they have. Both on the skills of what to do, but also the perception of the providers around the intervention in terms of buy-in of that task.*

#### Burdensome aspects of research participation

We asked participants to discuss the burdensome aspects of research participation. Three themes emerged across participants types:

1. **Adding to workload.** The extent to which research adds to an existing workload was raised as potentially burdensome aspect of staff participation in research. As one CHC staff member commented:
*So, when I hear the word burdensome when it comes to research, it’s the add on layer to the work that we do already. We are so overwhelmed. And then you have all these things waiting for you then they throw out research. And because there are very few Community Health Workers, we end up being involved in all this research.*

1. **Time burden.** Participants discussed the time and effort it takes to participate in research activities as a source of burden. As one CHC staff member commented:
*Burden comes up with time as you’re reprioritizing and budgeting your day or a period of time. None of us are in positions with the luxury of shaving time off of our responsibilities. It’s making the pile of responsibilities larger, which extends the clock.*

1. **Limited capacity and resources.** Participants discussed limited resources, research experience, and ability to conduct research at CHCs as a burden for supporting research. As one CHC staff member commented:
*When I think of burden, I think about the fact that health centers don’t have extra anything. We barely have enough to do our regular work. We don’t have extra people, we don’t have extra time, we don’t have extra rooms. So anytime a researcher says, ‘Don’t worry, it is not going to have any impact, no burden,’ it is never true.*

**Benefits of research participation.** We asked participants to share their thoughts on the benefits of research participation. Three themes emerged across participants types:

1. **Improve healthcare delivery and outcomes.** Participants believed one of the benefits of research participation is the potential to improve healthcare delivery and outcomes. As one researcher noted:
*Some of the work we’ve done is either the perceived benefit or the actual benefit of an improved workflow that maybe they didn’t have time for quality improvement purposes to test out on their own but we’re helping facilitate that.*

1. **Staff recruitment and retention.** Opportunities to be involved in research were highlighted by participants as a factor that might improve staff recruitment and retention. As one CHC staff participant commented:
*For staff, especially providers, they tend to have interest in research. They don’t necessarily have the time for it, but it’s a good recruitment tool because there’s an opportunity to do research and that looks nice for my career and that’s important to me.*

1. **Networking and relationship building.** CHC staff mentioned that participating in research can be a way to network with peers and colleagues, and researcher participants spoke about the opportunity for staff to build relationships with researchers, healthcare organizations, and community members through research activities. As one CHC staff commented:
*There’re opportunities like this one where you get to meet folks who do work that’s similar to yours from other settings, that’s a positive experience. And so we often have the opportunity to meet people again, and that has been beneficial to me.*

**Ways to improve research participation**

Finally, we asked participants to share their thoughts about ways to make it easier for staff working in resource-constrained healthcare settings to participate in research. Five themes emerged across both participants types:

1. **Transparency about the research process.** Participants spoke about the need for transparency in research, including the rationale for the research, explaining the data collection methods, and reviewing the study timeline at the outset. As one researcher commented:
*I think that upfront negotiation about doing research or not is important. If staff miss some of the big asks that researchers have and those come up later, staff feel like somebody sprung this stuff on them after they agreed to do this.*

1. **Organization and individual capacity building.** Participants discussed how research projects and initiatives that build and strengthen organizational and individual capacity to conduct research would promote staff engagement in research. As one CHC staff member commented:
*There’s a lot of folks that I work with that wouldn’t have experience with implementation science and if that’s important to the team, the researchers should transfer the knowledge about the methods they’re using to the health center. Without it, folks would be unsure why we’re calling it one thing or another. Method capacity building can help facilitate research because staff are more engaged in the research activities.*

1. **Apply user-centered design** Participants discussed the need to use methods and approaches to research that fit the people and setting. As one researcher commented:
*As a field, we have a lot of opportunities to be creative and flexible in the methods that we use to better align with kind of the needs and preferences of the folks that we’re doing research with and kind of being accepting of those things.*

1. **Offer equitable incentives for staff.** Participants emphasized the importance of offering equitable incentives, both financial and nonfinancial (e.g., social activities, recognition and praise, and food) for staff participation in research. As one CHC staff member commented:
*The other thing is kind of simple, rewards, food. We had a survey done here that was very important to the organization. And at one point, they created a drop-inspace with the idea of come in, have a cup of coffee, grab a donut while you’re here.*

1. **Bring more joy into research work.** Participants spoke about the need to make research participation fun and bring joy into research. As one CHC staff member commented:
*You should also recruit your champions or people that are the go-getters in the place that you’re going to be doing research where they can be excited to do a focus group. And for everyone to be excited, you have to garner excitement about it because if it’s not exciting, why do you want to do it?*

### Consensus discussions

As shown in Fig. 1, during the consensus discussions we mapped practical considerations for selected themes identified from the focus groups into different phases of the EPIS framework. For example, to align research with organizational and staff values and priorities, assessment of those values and priorities could occur in the Exploration phase. Co-designing the research with staff, in light of those values and priorities, could occur during the Planning phase. Monitoring research progress and its alignment with values and priorities could occur during Implementation, and sharing of results and examination of alignment could occur to advance Sustainment.

## Discussion

This qualitative study provides an in-depth exploration of the factors that influence staff participation in research, perceived research burdens and benefits, and ways to facilitate participation in research among staff working in CHCs and other resource-constrained healthcare settings. This study builds on two prior quantitative surveys of CHC’s capacity and readiness, perceived needs, and barriers and facilitators for research participation and collaborations [6,27] by partnering with CHC staff and researchers involved an implementation science network to identify practical considerations for engaging staff as participants and/or partners in research across different stages of implementation, from exploration to sustainment. Our study team developed scenario-based examples of how these considerations could be enacted in practice to address challenges and leverage facilitators to engagement in research. Future research should build on these qualitative findings to develop and test strategies and tools for engaging staff working in resource-constrained healthcare settings in implementation research.

The qualitative findings on barriers and facilitators to CHC staff participation in research from this study are similar to findings from a national survey of CHC participation in research conducted by Beeson and colleagues [27] and a statewide survey by Brandt and colleagues [6]. Across all three studies, CHCs reported dedicated staff time and concerns about loss of productivity as major barriers to research participation. With regard to perceived benefits of research participation, the potential to improve healthcare delivery and outcomes was a finding from our study that echoed findings from the Brandt et al study. In addition, all three studies found that CHC’s were interested in training opportunities and resources for research. The current study builds on prior research by identifying, with community partners, practical considerations for addressing barriers and leveraging facilitators to staff participation across different stages of implementation research. For example, we suggest that researchers offer CHC staff opportunities to partner during the exploration phase of implementation to identify areas for research capacity building and use a co-design approach during the preparation phase to create an action plan for strengthening human and organizational research capacity. During the implementation phase, research teams could partner with staff to monitor research progress and its continued alignment with organizational and individual values identified during the exploration phase. As the context in which EBIs are implemented is dynamic may change over time [28,29], it will be important to assess how well an EBI continues to align with values and priorities as a strategy for engaging and retaining staff in research. Finally, during the sustainment phase of implementation research, we suggest that research teams offer training and support for organizations and staff on implementation strategies and methods for ongoing evaluation of EBIs as an engagement strategy during this phase of research.

Research teams have developed processes and tools for assessing contextual factors for implementing EBIs [30–32] which informs tailoring of implementation strategies to the unique characteristics and circumstances of the setting where it is being implemented [33]. However, a methodological gap exists with respect to the availability of structured and systematic processes for assessing barriers and facilitators to engaging staff as participants and/or partners in implementation research and strategies and tools designed to address these contextual factors and promote engagement in research. This gap represents an opportunity to advance the science of engagement in implementation research. Future research should build on findings from the present qualitative study and prior studies on CHC participation in research [6,27] to test the effectiveness of methods for engaging CHC staff in implementation research. One potential method might be to use variations of cases generated by qualitative studies such as ours to engage researchers and staff in actively learning from different types of potential challenges, solutions, and successes before facing them in real time during the implementation.

Limitations of this study include focusing on selected themes applied to the EPIS framework and the potential limited generalizability of study findings to other resource-constrained healthcare settings. While it was not feasible in the current methods pilot to address all qualitative themes, our team generated practical considerations for the salient themes identified during consensus discussions and mapped them to the four phases of implementation. Consistent with qualitative research methods, the present study focused on depth of understanding and thematic saturation [34] that included a relatively small sample size of individual participants. In addition, it is important to note that this study took place in CHCs that are considered resource-constrained settings in the U.S. healthcare context. While the themes (e.g., align research with organizational and individual priorities and values) may apply to resource-constrained settings in the global healthcare context, the practical considerations and scenario-based examples may not be generalizable to global settings.

## Conclusions

Engaging staff in resource-constrained settings as participants and partners in implementation research requires knowledge about what contributes to research burden and benefits. Implementation research likely involves some level of burden for staff. An implementation science framework can help facilitate planning to address context-specific burdens and leverage perceived benefits to promote staff engagement in research. The mapping of practical considerations for engaging staff in implementation research to the phases of EPIS may help facilitate discussion and action planning in various stages of implementation research. Future research should build on findings from the present qualitative study and other prior related research to test the effectiveness of methods for engaging staff working in resource-constrained healthcare settings in implementation research.

## Supporting information

Aschbrenner et al. supplementary material 1 Aschbrenner et al. supplementary material

Aschbrenner et al. supplementary material 2 Aschbrenner et al. supplementary material

Aschbrenner et al. supplementary material 3 Aschbrenner et al. supplementary material

Aschbrenner et al. supplementary material 4 Aschbrenner et al. supplementary material

## Supplementary material

The supplementary material for this article can be found at https://doi.org/10.1017/cts.2025.29.
