# Supplementary material for: Practical considerations for engaging staff in resource-constrained healthcare settings in implementation research: A qualitative focus group and consensus building study
Source: J Clin Transl Sci. 2025 Mar 26;9(1):e65. doi: 10.1017/cts.2025.29 (PMC11975774; doi:10.1017/cts.2025.29)
Supplement: Aschbrenner et al. supplementary material 1 — Aschbrenner et al. supplementary material [file S2059866125000299sup001.docx]

**Supplemental File 1.**

***Theme 1: Align research with organizational and staff values and priorities.***

**Scenario-based example:** Dr. Jones wants to study implementation strategies for bundling evidence-based cancer screening interventions in a community health center (CHC). Bundling involves combining evidence-based interventions to improve implementation, client or service outcomes and/or enhance behavioral or health outcomes for multiple cancer types or other health concerns (1). Given the growing number of evidence-based cancer screening interventions (2), Dr. Jones will need to work with healthcare partners to select the evidence-based interventions for integration in community health centers. An important part of this process will be aligning the bundled interventions with organization and staff values and priorities. Dr. Jones partners with CHC leaders and staff to align the planned research with organizational and staff values and priorities using Exploration, Preparation, Implementation, Sustainment (EPIS) (3) as a framework for thinking through alignment at different phases of the implementation research.

**Exploration:** With input from CHC partners, Dr. Jones assesses priorities for bundling evidence-based cancer screening interventions by conducting a brief online survey asking leadership and staff at multiple levels to rate the extent to which certain evidence-based cancer screening interventions are prioritized and valued. She follows up the survey results with a series of focus groups with leadership and staff to further understand why certain evidence-based cancer screening interventions are prioritized and valued over others, and to assess their preferences for bundling individual interventions. Dr. Jones works closely with her CHC partners to consider priorities and values as a key factor in the selection of the bundled screening intervention.

**Preparation**: Dr. Jones uses co-design principles to plan for the implementation of the bundled evidence-based screening interventions and the associated research activities (e.g., data collection) to make sure they are aligned with the identified organizational and staff values and priorities. Co-design is a collaborative approach where researchers and partners work together to develop solutions to intervention and/or implementation challenges (4). Dr. Jones recruits a diverse group of staff from within the CHC to provide specific input on plans for an implementation strategy for the bundled screening intervention. She also seeks their input on the practicality of the data collection methods (e.g., online surveys and interviews) that will be used in the study.

**Implementation:** Recognizing that organization and staff values and priorities for bundled cancer screening interventions may change over the 5-year study, Dr. Jones works with her CHC partners to develop a plan for ongoing monitoring of the implementation of the bundled cancer screening intervention relative to organization and staff values and priorities. The plan includes holding twice yearly meetings with leadership and staff to seek continuous input throughout the study period to inform modifications to the intervention and/or implementation strategy to better align with changing priorities and values. If modifications are necessary, Dr. Jones and her team will guide the modifications using established implementation science frameworks (5, 6), ensuring that fidelity to the core components of the implementation and/or intervention are maintained.

**Sustainment:**  During the final year of the study, Dr. Jones plans to share preliminary results including process and outcomes with CHC leadership and staff to determine whether the research advanced organization and staff values and priorities as intended. Based on these findings and current priorities, values, and preferences for cancer screening interventions, Dr. Jones will work with the CHC team to design a sustainability plan for continuing the bundled screening intervention in routine practice once the study has ended. The plan will be informed by the Clinical Sustainability Assessment Tool (CSAT)(7), a self-assessment used by both clinical staff and partners to evaluate the sustainability capacity of a clinical practice. Users of the tool receive a free sustainability report that summarizes and calculates a score to use in sustainability planning. The tool will be completed midway through the study and the informed used to plan for long-term sustainment.

**References:**

1. Villalobos A, Chambers DA. Advancing the science of integrating multiple interventions by blending and bundling. JNCI Cancer Spectr. 2023;7(5).

2. Rintala S, Dahlstrom KR, Franco EL, Louvanto K. A synthesis of evidence for cancer-specific screening interventions: A Preventive Medicine Golden Jubilee Review. Preventive Medicine. 2023;167:107395.

3. Aarons GA, Hurlburt M, Horwitz SM. Advancing a conceptual model of evidence-based practice implementation in public service sectors. Adm Policy Ment Health. 2011;38(1):4-23.

4. Vargas C, Whelan J, Brimblecombe J, Allender S. Co-creation, co-design, co-production for public health - a perspective on definition and distinctions. Public Health Res Pract. 2022;32(2).

5. Wiltsey Stirman S, Baumann AA, Miller CJ. The FRAME: an expanded framework for reporting adaptations and modifications to evidence-based interventions. Implement Sci. 2019;14(1):58.

6. Kirk MA, Haines ER, Rokoske FS, Powell BJ, Weinberger M, Hanson LC, et al. A case study of a theory-based method for identifying and reporting core functions and forms of evidence-based interventions. Transl Behav Med. 2021;11(1):21-33.

7. Malone S, Prewitt K, Hackett R, Lin JC, McKay V, Walsh-Bailey C, et al. The Clinical Sustainability Assessment Tool: measuring organizational capacity to promote sustainability in healthcare. Implementation Science Communications. 2021;2(1):77.
