# Supplementary material for: Practical considerations for engaging staff in resource-constrained healthcare settings in implementation research: A qualitative focus group and consensus building study
Source: J Clin Transl Sci. 2025 Mar 26;9(1):e65. doi: 10.1017/cts.2025.29 (PMC11975774; doi:10.1017/cts.2025.29)
Supplement: Aschbrenner et al. supplementary material 2 — Aschbrenner et al. supplementary material [file S2059866125000299sup002.docx]

**Supplemental File 2.**

***Theme 2: Apply user-centered design to research methods and approaches***

**Scenario-based example:** Dr. Lee plans to conduct a mixed methods study that uses a combination of online surveys, interviews, focus groups, and observations to collect data on perceived barriers and facilitators to implementing evidence-based cancer prevention interventions in community health centers (CHCs). While the study design is rigorous and follows guidance from seminal textbooks [1], Dr. Lee knows from prior experience that getting input from end users on the methods and approaches used to conduct research is critical for promoting participation in the research. She understands that staff want researchers to consider the workloads, workflows, and research experience and capacity of staff and the CHC when planning the research methods. Dr. Lee forms an advisory group that includes CHC leaders and staff to explore, prepare, and implement the study.

**Exploration:** Dr. Lee presents the proposed mixed methods design to the CHC advisory team to get input. To accommodate staff with varying degrees of familiarity in research, Dr. Lee prepares a brief lay summary of key terms used throughout the presentation. The following is a list of example questions that Dr. Lee presents to the advisory team:

- *How realistic is the planned sample size for the study?*
- *How feasible is it to conduct online surveys, interviews, focus groups, and observations with staff?*
- *To what extent are the incentives appealing, appropriate and equitable for staff?*
- *How realistic is the study timeline?*
- *Are there any changes you recommend to the study design?*
- *Who are candidates for internal champions at the CHC who will promote the study to staff?*
- *Are there any space and/or technology requirements to consider?*

**Preparation**: During the preparation phase, Dr. Lee works closely with CHC collaborators to think through and refine the methods used in this study. She incorporates a user-centered design approach [2], seeking feedback from the end users who will ultimately participate in the research. She pilots the online survey to a small group of five staff members to get input on the questions and format. She asks the advisory group to read a draft of the interview guide to spot any questions that participants may have difficulty answering, and suggest more relevant terms for unfamiliar jargon. Dr. Lee also asks the advisory group to review the structured protocol she developed for the observations to ensure the observations will not disrupt clinic workflows. The advisory group participants are provided monetary compensation for their time spent in meetings and reviewing materials.

**Implementation:** During the implementation of the mixed methods study, Dr. Lee has a brief weekly email check-in with the CHC internal champions, and meets monthly with the advisory group to monitor research activities and address any concerns as they arise. Dr. Lee leaves her contact information with study participants in case they have any follow-up thoughts they want to share about the research topic and methods. During this phase, Dr. Lee learns some staff have difficulty participating in interviews as it is disruptive to be removed from patient care. She brainstorms solutions with the advisory group, and the decision is made to reconfigure research team schedules to accommodate interview slots before and after clinic hours to accommodate staff who cannot participate during patient care hours.

**Sustainment:** Through the course of conducting this mixed methods study, Dr. Lee learns that the quality improvement team at the CHC is interested in learning how to collect and integrate quantitative and qualitive data to evaluate quality improvement processes. In response, Dr. Lee hosts a free, 2-hour workshop on mixed methods study design, data collection, data analysis, and interpretation for the quality improvement team and any other CHC staff who were interested in an introduction to mixed methods research. Dr. Lee provides the workshop recording and informational handouts via a free, cloud-based storage platform for future access.

**References:**

1. Creswell JW, Clark VLP. Designing and Conducting Mixed Methods Research: SAGE Publications; 2011.

2. Dopp AR, Parisi KE, Munson SA, Lyon AR. A glossary of user-centered design strategies for implementation experts. Translational Behavioral Medicine. 2018;9(6):1057-64.
