# Supplementary material for: Practical considerations for engaging staff in resource-constrained healthcare settings in implementation research: A qualitative focus group and consensus building study
Source: J Clin Transl Sci. 2025 Mar 26;9(1):e65. doi: 10.1017/cts.2025.29 (PMC11975774; doi:10.1017/cts.2025.29)
Supplement: Aschbrenner et al. supplementary material 3 — Aschbrenner et al. supplementary material [file S2059866125000299sup003.docx]

**Supplemental File 3.**

***Theme 3*: *Build organizational and individual research capacity.***

**Scenario-based example:** Dr. Brown is collaborating with a community health center (CHC) to develop and pilot test an implementation strategy for colorectal cancer (CRC) screening. The Partnered and Equity Data-Driven Implementation (PEDDI) strategy [1] involves an external facilitator who guides interactive problem solving and provides support to an internal team of healthcare professionals responsible for implementing the CRC screening intervention [2]. The facilitator guides the CHC team to obtain and use data collected in routine clinical practice to: (a) identify patient groups experiencing gaps in outreach, use, and/or benefit from CRC screening, and (b) rapidly adapt screening outreach and/or the CRC screening intervention to address identified gaps. As part of the implementation strategy development process, Dr. Brown uses a user-centered design methodology [3] to evaluate the usability of the implementation strategy and generate solutions to address the identified usability issues [4]. Dr. Brown learns that the quality improvement and population health team wants training in the user-centered design methodology used in this pilot study. She collaborates with CHC partners to explore, prepare, implement and sustain capacity building for the methodology.

**Exploration:** Guided by a framework for planning and measuring research capacity building in practice settings [5], Dr. Brown and her team assess current and desired skills, knowledge and resources for user-centered design methodology at the individual and organization level. Given the time and funding constraints of the pilot study, Dr. Brown and her CHC partners focus on what specific skills could be developed and strengthened in the context of the pilot study. The team conducts a needs assessment at the organizational level that includes assessing the availability and use of professional development funds or protected time for staff to participate in user-centered design trainings; levels of existing skills within the workforce; and opportunity to match novice staff with staff experienced in user-centered design methodology. The team also assesses individual staff interest in and experience with user-centered design methodologies; availability to participate in training; and confidence that they could share new skills and knowledge with others and apply the skills in practice.

**Preparation**: Dr. Brown works closely with CHC partners to co-design a plan for strengthening individual and organizational capacity for user-centered design methodology. The CHC asks Dr. Brown to provide education and facilitate skills practice in the user-centered design method. To accommodate schedules, the training on the desired methods and skills practice is planned to be delivered across 6 weekly lunch and learn sessions supplemented by an online learning platform hosted by Dr. Brown’s university during the pilot study period.

**Implementation:** Dr. Brown and her CHC partners develop a plan to monitor and evaluate progress toward user-centered design methodology capacity building goals and objectives. The plan includes conducting brief online evaluations after each of the 6 lunch and learn sessions to assess the knowledge acquisition and perceived skills development. In addition, Dr. Brown meets with the quality improvement and population health staff teams every 2 weeks during the 6 week period to assess the extent to which staff are developing skills in the training, and integrates feedback to modify the training activities to better meet learner needs. She also observes team members applying the skills in practice during the final 2 weeks of the series.

**Sustainment:** Dr. Brown and her CHC partners identify high performing staff to take on the role of trainers in the user-centered design methods so the capacity building efforts could continue once the pilot study ends. Dr. Brown uses a Train-the-Trainer Model to prepare two staff members as master trainers to train other staff in the method method and apply these in the context of quality improvement and population health initiatives

**References:**

1. Aschbrenner KA, Kruse G, Emmons KM, Singh D, Barber-Dubois ME, Miller AM, et al. Stakeholder and Equity Data-Driven Implementation: a Mixed Methods Pilot Feasibility Study. Prev Sci. 2022:1-11.

2. Stetler CB, Legro MW, Rycroft-Malone J, Bowman C, Curran G, Guihan M, et al. Role of "external facilitation" in implementation of research findings: a qualitative evaluation of facilitation experiences in the Veterans Health Administration. Implement Sci. 2006;1:23.

3. Lyon AR, Coifman J, Cook H, McRee E, Liu FF, Ludwig K, et al. The Cognitive Walkthrough for Implementation Strategies (CWIS): a pragmatic method for assessing implementation strategy usability. Implement Sci Commun. 2021;2(1):78.

4. Aschbrenner KA, Haines ER, Kruse GR, Olugbenga AO, Thomas AN, Khan T, et al. Applying cognitive walkthrough methodology to improve the usability of an equity-focused implementation strategy. Implementation Science Communications. 2024;5(1):95.

5. Cooke J. A framework to evaluate research capacity building in health care. BMC Fam Pract. 2005;6:44.
