# Supplementary material for: Practical considerations for engaging staff in resource-constrained healthcare settings in implementation research: A qualitative focus group and consensus building study
Source: J Clin Transl Sci. 2025 Mar 26;9(1):e65. doi: 10.1017/cts.2025.29 (PMC11975774; doi:10.1017/cts.2025.29)
Supplement: Aschbrenner et al. supplementary material 4 — Aschbrenner et al. supplementary material [file S2059866125000299sup004.docx]

**Supplemntal File 4.**

***Theme 4*: *Offer equitable incentives for staff participation.***

**Scenario-based example:** Dr. Ross plans to conduct a mixed methods study that uses a combination of online surveys and semi-structured interviews to assess barriers and facilitators to implementing an evidence-based intervention (EBI) in community health centers (CHCs). Dr. Ross plans to survey and interview the chief operating officer; medical director; director of quality improvement; director of operations; population health manager and staff; quality improvement manager and staff; and primary care providers (including physicians, physician assistants, and nurse practitioners). She knows from prior research partnerships it is important to develop an equitable plan for monetary and non-monetary incentives for staff who participate in research. Dr. Ross collaborates with her CHC partners to plan incentives for participation in the mixed methods study.

**Exploration:** Dr. Ross and her CHC partners discuss monetary and non-monetary incentives for research participation. A key recommendation for recruiting healthcare professionals for research is to identify ways that they can be recognized by organizational leaders for their time and effort participating in research [1]. Dr. Ross learns that many staff want opportunities to contribute to products from research, including scientific manuscripts and conference presentations. She plans to invite CHC staff to co-author manuscripts and co-present study findings at a local professional meeting. Regarding monetary incentives, all staff who complete study activities, regardless of their seniority and/or position or role at the CHC, are provided the same cash value compensation. The plan for both monetary and non-monetary incentives is done in alignment with research ethics and IRB approval.

**Preparation**: Dr. Ross collaborates with her CHC partners to plan the implementation of the incentives for participation in the study. They work together to craft emails and create advertisements that communicate the incentives in ways that are appealing to CHC staff. Dr. Ross and her research team also get input on how to efficiently award the monetary incentives to staff immediately following their completion of the study. They agree to send electronic gift cards to staff via email within 24 hours of completing an online survey or interview.

**Implementation:** Throughout the implementation of the mixed methods study, Dr. Ross asks CHC partners to provide feedback about the acceptability and strength of incentives for staff participation in research. She monitors the distribution of incentives weekly by reviewing a tracking sheet prepared by the study coordinator to make sure the incentives are awarded on a timely basis. She also tracks the contact information of participants who express interest in co-authoring manuscripts and/or co-presenting study findings.

**Sustainment:** Dr. Ross collaborates with her CHC partners to explore whether aspects of the incentive structure and/or processes developed in this study could be sustained to support future research led by her and others. As a result of this study, the CHC now has a written protocol regarding equitable incentives for staff participation in research to use as a starting point in future research. Finally, Dr. Ross and her team host online writing sessions, offered several times per week, for CHC co-authors to join to collaborate on manuscript writing and preparing presentations. Dr. Ross leverages grant writing support from her institution to apply for scholarship applications with CHC partners to cover the cost of CHC staff co-presenter attendance at professional meetings.

**References:**

1. Browne S, Dooley S, Geraghty A, Dominguez Castro P, Reynolds C, Perrotta C, et al. Reflections on recruiting healthcare professionals as research participants: Learning from the ONSPres Study. HRB Open Res. 2022;5:47.
